# Supplementary material for: The Mus musculus Papillomavirus Type 1 E7 Protein Binds to the Retinoblastoma Tumor Suppressor: Implications for Viral Pathogenesis
Source: mBio. 2021 Aug 31;12(4):e02277-21. doi: 10.1128/mBio.02277-21 (PMC8406179; doi:10.1128/mBio.02277-21)
Supplement: TABLE S1 [file mbio.02277-21-st001.docx]

**Table S1.** Cellular proteins identified by Affinity purification/mass spectrometry (AP/MS) analyses of carboxyl- or amino terminally epitope tagged MmuPV1 E7 (CE7 and NE7 respectively). The number of unique and total peptides identified for each protein are shown. See reference [17] for experimental details.

|  | **MmuPV1 CE7** |  |  |  | **MmuPV1 NE7** |  |
| --- | --- | --- | --- | --- | --- | --- |
| **unique** | **total** | **gene** |  | **gene** | **unique** | **total** |
|  |  |  |  | AAR2 | 6 | 6 |
|  |  |  |  | ABT1 | 3 | 3 |
|  |  |  |  | ACOX1 | 5 | 5 |
| 5 | 5 | AGGF1 |  | AGGF1 | 9 | 10 |
| 3 | 5 | ALMS1 |  |  |  |  |
| 9 | 9 | ANAPC1 |  | ANAPC1 | 36 | 48 |
|  |  |  |  | ANAPC10 | 5 | 8 |
|  |  |  |  | ANAPC16 | 4 | 8 |
|  |  |  |  | ANAPC2 | 8 | 8 |
| 5 | 8 | ANAPC4 |  | ANAPC4 | 15 | 19 |
| 5 | 7 | ANAPC5 |  | ANAPC5 | 20 | 25 |
|  |  |  |  | ANAPC7 | 11 | 11 |
| 11 | 11 | ARHGAP35 |  | ARHGAP35 | 34 | 42 |
|  |  |  |  | ARHGEF10 | 14 | 17 |
| 3 | 4 | ARHGEF10L |  | ARHGEF10L | 19 | 21 |
| 41 | 71 | ARHGEF2 |  | ARHGEF2 | 19 | 25 |
|  |  |  |  | ARHGEF40 | 8 | 9 |
|  |  |  |  | ARMCX3 | 4 | 5 |
| 12 | 15 | ARVCF |  | ARVCF | 28 | 50 |
| 5 | 6 | ASPM |  | ASPM | 11 | 11 |
|  |  |  |  | BARD1 | 11 | 12 |
| 5 | 5 | BCR |  | BCR | 18 | 19 |
|  |  |  |  | BDP1 | 3 | 3 |
|  |  |  |  | BOP1 | 9 | 12 |
|  |  |  |  | BRCA1 | 27 | 34 |
| 11 | 16 | C2CD2 |  | C2CD2 | 7 | 9 |
| 4 | 4 | C2CD2L |  | C2CD2L | 3 | 3 |
|  |  |  |  | C5orf45 | 3 | 3 |
|  |  |  |  | CAB39 | 3 | 3 |
| 9 | 10 | CAMSAP1 |  | CAMSAP1 | 30 | 37 |
| 11 | 14 | CAMSAP2 |  | CAMSAP2 | 25 | 35 |
|  |  |  |  | CASK | 16 | 18 |
|  |  |  |  | CCNG1 | 4 | 4 |
| 3 | 3 | CDC123 |  | CDC123 | 3 | 3 |
| 5 | 7 | CDC16 |  | CDC16 | 14 | 21 |
| 8 | 9 | CDC23 |  | CDC23 | 14 | 16 |
| 3 | 3 | CDC26 |  | CDC26 | 3 | 6 |
| 11 | 11 | CDC27 |  | CDC27 | 21 | 28 |
|  |  |  |  | CDK11A | 4 | 4 |
|  |  |  |  | CDK5 | 5 | 5 |
| 4 | 5 | CDK7 |  | CDK7 | 10 | 11 |
|  |  |  |  | CDYL | 4 | 4 |
| 19 | 29 | CHAMP1 |  | CHAMP1 | 38 | 53 |
|  |  |  |  | CHTF18 | 3 | 3 |
| 3 | 4 | CRTC3 |  | CRTC3 | 3 | 3 |
| 7 | 8 | CSNK1A1 |  | CSNK1A1 | 6 | 7 |
| 4 | 6 | CSNK1A1L |  | CSNK1A1L | 4 | 5 |
| 3 | 3 | CSNK1D |  | CSNK1D | 5 | 6 |
|  |  |  |  | DAP3 | 9 | 9 |
|  |  |  |  | DAPK3 | 5 | 6 |
| 7 | 7 | DCAF13 |  | DCAF13 | 8 | 9 |
| 4 | 5 | DCP2 |  | DCP2 | 3 | 3 |
|  |  |  |  | DENND2C | 8 | 9 |
|  |  |  |  | DIEXF | 18 | 18 |
|  |  |  |  | DLG1 | 11 | 13 |
| 12 | 21 | DONSON |  | DONSON | 14 | 23 |
| 3 | 3 | ECD |  |  |  |  |
|  |  |  |  | EHMT2 | 6 | 6 |
|  |  |  |  | ELP2 | 4 | 4 |
| 4 | 4 | ELP3 |  | ELP3 | 13 | 14 |
| 20 | 47 | ELP4 |  | ELP4 | 22 | 55 |
| 6 | 10 | ELP5 |  | ELP5 | 6 | 13 |
| 13 | 21 | ELP6 |  | ELP6 | 16 | 40 |
| 10 | 10 | EML3 |  |  |  |  |
|  |  |  |  | ERCC6L | 21 | 25 |
|  |  |  |  | ERN1 | 9 | 9 |
| 7 | 8 | ESCO2 |  |  |  |  |
| 4 | 4 | ESF1 |  | ESF1 | 19 | 23 |
|  |  |  |  | ETV6 | 6 | 7 |
|  |  |  |  | EXPH5 | 4 | 4 |
|  |  |  |  | FAM135A | 6 | 7 |
|  |  |  |  | FAM192A | 4 | 6 |
| 5 | 5 | FAM207A |  |  |  |  |
|  |  |  |  | FAM83B | 4 | 4 |
| 9 | 11 | FAM83G |  | FAM83G | 14 | 17 |
| 4 | 4 | FBXO11 |  |  |  |  |
| 11 | 20 | FRMD5 |  | FRMD5 | 13 | 22 |
| 12 | 13 | FRMD6 |  | FRMD6 | 11 | 17 |
|  |  |  |  | FRYL | 10 | 13 |
|  |  |  |  | FTSJ3 | 3 | 3 |
|  |  |  |  | FZR1 | 4 | 4 |
| 3 | 3 | GID8 |  | GID8 | 4 | 4 |
| 4 | 4 | GNA11 |  |  |  |  |
|  |  |  |  | GPATCH2L | 3 | 3 |
| 3 | 6 | GRWD1 |  | GRWD1 | 6 | 8 |
|  |  |  |  | GSR | 4 | 4 |
| 6 | 8 | GTF2B |  | GTF2B | 6 | 7 |
|  |  |  |  | GTF2F1 | 3 | 3 |
|  |  |  |  | GTF3C1 | 6 | 7 |
|  |  |  |  | GTF3C3 | 5 | 6 |
|  |  |  |  | GTF3C4 | 4 | 4 |
| 4 | 5 | GULP1 |  | GULP1 | 6 | 7 |
|  |  |  |  | HBS1L | 8 | 10 |
|  |  |  |  | HDAC4 | 3 | 3 |
|  |  |  |  | HDAC6 | 3 | 3 |
|  |  |  |  | HEXIM1 | 3 | 3 |
|  |  |  |  | HIRA | 3 | 3 |
|  |  |  |  | HJURP | 6 | 9 |
| 12 | 15 | IBTK |  | IBTK | 45 | 69 |
|  |  |  |  | ILVBL | 3 | 3 |
| 23 | 33 | INF2 |  | INF2 | 24 | 31 |
|  |  |  |  | JMJD1C | 3 | 3 |
|  |  |  |  | KDELC1 | 4 | 4 |
|  |  |  |  | KDM3B | 3 | 3 |
| 3 | 3 | KLC1 |  | KLC1 | 7 | 7 |
|  |  |  |  | KSR2 | 4 | 4 |
|  |  |  |  | LENG8 | 4 | 4 |
|  |  |  |  | LIMK1 | 3 | 3 |
|  |  |  |  | LIN7A | 3 | 3 |
|  |  |  |  | LLGL2 | 3 | 4 |
| 6 | 6 | LRWD1 |  | LRWD1 | 3 | 4 |
| 4 | 4 | LSG1 |  | LSG1 | 6 | 6 |
|  |  |  |  | LUC7L2 | 3 | 3 |
|  |  |  |  | MAML3 | 3 | 4 |
|  |  |  |  | MAP2K4 | 3 | 3 |
|  |  |  |  | MAPKAP1 | 4 | 4 |
|  |  |  |  | MAX | 3 | 3 |
|  |  |  |  | MPHOSPH10 | 3 | 4 |
|  |  |  |  | MPHOSPH9 | 7 | 8 |
| 3 | 4 | MRE11A |  | MRE11A | 4 | 4 |
|  |  |  |  | MSL1 | 8 | 11 |
| 3 | 3 | MTBP |  | MTBP | 14 | 15 |
|  |  |  |  | MTDH | 3 | 3 |
| 4 | 4 | NEDD4 |  |  |  |  |
| 6 | 7 | NET1 |  | NET1 | 4 | 4 |
|  |  |  |  | NFXL1 | 3 | 3 |
|  |  |  |  | NKRF | 3 | 3 |
|  |  |  |  | NOA1 | 3 | 3 |
|  |  |  |  | NPHP4 | 8 | 8 |
|  |  |  |  | NR1D1 | 7 | 7 |
|  |  |  |  | NUB1 | 3 | 3 |
| 3 | 3 | NUMB |  | NUMB | 8 | 8 |
|  |  |  |  | ODF2 | 11 | 13 |
|  |  |  |  | ORC3 | 4 | 5 |
|  |  |  |  | OXSR1 | 3 | 5 |
| 3 | 3 | PAK4 |  |  |  |  |
| 16 | 22 | PAK6 |  | PAK6 | 19 | 26 |
|  |  |  |  | PATL1 | 4 | 4 |
| 3 | 3 | PAWR |  |  |  |  |
|  |  |  |  | PES1 | 4 | 4 |
|  |  |  |  | PEX1 | 4 | 4 |
|  |  |  |  | PHRF1 | 12 | 18 |
| 3 | 3 | PIK3C2A |  |  |  |  |
| 4 | 5 | PIKFYVE |  | PIKFYVE | 11 | 11 |
| 10 | 10 | PKN2 |  | PKN2 | 13 | 15 |
| 3 | 3 | POGZ |  | POGZ | 14 | 18 |
|  |  |  |  | POP1 | 3 | 3 |
| 3 | 34 | POTEKP |  |  |  |  |
|  |  |  |  | PRKAA1 | 5 | 5 |
|  |  |  |  | PRR14L | 7 | 9 |
| 3 | 3 | PSPC1 |  |  |  |  |
|  |  |  |  | PTCD3 | 9 | 10 |
|  |  |  |  | PTPN14 | 12 | 13 |
|  |  |  |  | PTPN21 | 13 | 16 |
| 17 | 85 | PTRF |  | PTRF | 12 | 22 |
| 9 | 11 | PUM1 |  | PUM1 | 14 | 16 |
| 7 | 7 | QSER1 |  | QSER1 | 21 | 26 |
| 9 | 12 | RACGAP1 |  |  |  |  |
|  |  |  |  | RAD50 | 4 | 5 |
|  |  |  |  | RAE1 | 3 | 3 |
| 16 | 22 | RB1 |  | RB1 | 34 | 82 |
|  |  |  |  | RBBP8 | 3 | 4 |
| 6 | 6 | RECQL4 |  | RECQL4 | 10 | 10 |
|  |  |  |  | RECQL5 | 5 | 5 |
| 3 | 3 | RELL1 |  | RELL1 | 6 | 9 |
| 4 | 4 | RHBDF1 |  | RHBDF1 | 3 | 4 |
|  |  |  |  | RPS6KA4 | 9 | 11 |
| 16 | 17 | RPS6KA5 |  | RPS6KA5 | 32 | 51 |
| 5 | 8 | RPS6KB1 |  | RPS6KB1 | 4 | 5 |
| 3 | 3 | RRP1B |  | RRP1B | 13 | 15 |
| 3 | 6 | RTN4 |  |  |  |  |
| 3 | 4 | SAP30BP |  | SAP30BP | 5 | 5 |
|  |  |  |  | SBNO1 | 3 | 3 |
|  |  |  |  | SECISBP2L | 6 | 7 |
| 5 | 7 | SENP5 |  | SENP5 | 7 | 7 |
| 8 | 9 | SENP6 |  | SENP6 | 31 | 42 |
| 7 | 8 | SETD2 |  | SETD2 | 39 | 47 |
| 8 | 8 | SETD5 |  | SETD5 | 23 | 32 |
|  |  |  |  | SH3BP4 | 6 | 8 |
|  |  |  |  | STAU1 | 5 | 5 |
|  |  |  |  | STIM2 | 6 | 8 |
| 3 | 3 | STK11 |  | STK11 | 5 | 7 |
|  |  |  |  | STRADA | 5 | 6 |
|  |  |  |  | SUCO | 12 | 12 |
| 22 | 27 | SUGP2 |  | SUGP2 | 13 | 15 |
|  |  |  |  | SYDE2 | 16 | 18 |
|  |  |  |  | TAB1 | 8 | 8 |
|  |  |  |  | TAB2 | 4 | 4 |
|  |  |  |  | TCF25 | 6 | 6 |
| 6 | 6 | TES |  |  |  |  |
| 6 | 7 | TFAM |  | TFAM | 11 | 13 |
| 9 | 11 | TFB2M |  | TFB2M | 14 | 19 |
|  |  |  |  | TGIF1 | 3 | 3 |
|  |  |  |  | TGS1 | 3 | 5 |
|  |  |  |  | TIAL1 | 3 | 3 |
| 16 | 18 | TICRR |  | TICRR | 35 | 39 |
|  |  |  |  | TIGD5 | 5 | 5 |
|  |  |  |  | TMEM33 | 3 | 3 |
| 18 | 26 | TNRC6A |  | TNRC6A | 12 | 17 |
| 3 | 3 | TOLLIP |  |  |  |  |
| 16 | 17 | TOPBP1 |  | TOPBP1 | 31 | 38 |
| 3 | 3 | TSG101 |  |  |  |  |
|  |  |  |  | TTF1 | 4 | 4 |
|  |  |  |  | TTLL4 | 5 | 5 |
|  |  |  |  | TYK2 | 4 | 4 |
| 13 | 15 | UBR4 |  |  |  |  |
| 13 | 14 | UBR5 |  | UBR5 | 40 | 48 |
|  |  |  |  | USO1 | 4 | 4 |
| 11 | 13 | USP43 |  | USP43 | 15 | 18 |
| 6 | 6 | UTP14A |  |  |  |  |
|  |  |  |  | VAC14 | 8 | 8 |
|  |  |  |  | VRK1 | 5 | 5 |
|  |  |  |  | WDR12 | 4 | 5 |
| 9 | 9 | WDR47 |  | WDR47 | 21 | 35 |
|  |  |  |  | WEE1 | 3 | 3 |
|  |  |  |  | WWC1 | 8 | 9 |
|  |  |  |  | WWC3 | 9 | 11 |
| 3 | 3 | YES1 |  |  |  |  |
|  |  |  |  | YTHDF2 | 3 | 3 |
|  |  |  |  | ZBTB33 | 7 | 9 |
|  |  |  |  | ZNF215 | 4 | 4 |
| 5 | 5 | ZNF295 |  | ZNF295 | 5 | 5 |
|  |  |  |  | ZNF318 | 6 | 6 |
| 3 | 3 | ZNF638 |  | ZNF638 | 30 | 37 |
|  |  |  |  | ZNF770 | 4 | 4 |
| 5 | 7 | ZNHIT6 |  | ZNHIT6 | 12 | 13 |
