# Supplementary material for: The Mus musculus Papillomavirus Type 1 E7 Protein Binds to the Retinoblastoma Tumor Suppressor: Implications for Viral Pathogenesis
Source: mBio. 2021 Aug 31;12(4):e02277-21. doi: 10.1128/mBio.02277-21 (PMC8406179; doi:10.1128/mBio.02277-21)
Supplement: TABLE S2 [file mbio.02277-21-st002.docx]

**Table S2:** Sequences of PCR primers used in this sudy

| Target gene | | Sequence |
| --- | --- | --- |
|  |  |  |
| Human qPCR |  |  |
| CCNE2 | FWD | 5'-CTATTTGGCTATGCTGGAGG |
|  | REV | 5'-TCTTCGGTGGTGTCATAATG |
| MCM2 | FWD | 5'-TGTCACCTGCTCTGCCACTAA |
|  | REV | 5'-GCAGCATGCGCAAGACTTT |
| PCNA | FWD | 5'-CCATCCTCAAGAAGGTGTTGG |
|  | REV | 5'-GTGTCCCATATCCGCAATTTTAT |
| MCM7 | FWD | 5'-CAGAGACCAGCAGATGTGATATT |
|  | REV | 5'-GGTGTGAAGCCACGAGATAC |
| GAPDH | FWD | 5'-GATTCCACCCATGGCAAATCC |
|  | REV | 5'-TGGGATTTCCATTGATGACAAG |
|  |  |  |
| Mus qPCR |  |  |
| MCM7 | FWD | 5'-GAGGCCAGCAGATGTGATATT |
|  | REV | 5'-GGTGTGAAGCCACGAGATATG |
| CCNE2 | FWD | 5'-ATTTGGCTTTGCTGAATGAAGT |
|  | REV | 5'-CAGTACTCTTTGGTGGTGTCATA |
| MCM2 | FWD | 5'-CGGAGTATGCGCAAGACTTT |
|  | REV | 5'-GCCACCAACTGCTTCAGTAT |
| PCNA | FWD | 5'-GTTGTCACAAACAAGTAATGTGGAT |
|  | REV | 5'-CTCAGAGCAAACGTTAGGTGAA |
| GAPDH | FWD | 5'-GGAGAGTGTTTCCTCGTCCC |
|  | REV | 5'-ACTGTGCCGTTGAATTTGCC |
